# PABPN1 regulates mRNA alternative polyadenylation to inhibit bladder cancer progression

**DOI:** 10.1186/s13578-023-00997-6

**Published:** 2023-03-06

**Authors:** Liang Chen, Wei Dong, Menghao Zhou, Chenlu Yang, Ming Xiong, Gallina Kazobinka, Zhaohui Chen, Yifei Xing, Teng Hou

**Affiliations:** 1 Department of Urology, Union Hospital, Tongji Medical College, Huazhong University of Science and Technology, Wuhan, 430022 China; 2 Department of Gynecology and Obstetrics, Women and Children Hospital of Guangdong Province, Guangzhou, 510080 China; 3 Urology Unit, La Nouvelle Polyclinique Centrale de Bujumbura, Bujumbura, 378 Burundi; 4 Department of Urology, South China Hospital, Medical School, Shenzhen University, Shenzhen, 518116 China

**Keywords:** PABPN1, Bladder cancer, Alternative polyadenylation, Wnt signaling, Cell cycle, Lipid biosynthesis

## Abstract

**Background:**

About 10–20% of patients with bladder cancer (BC) progress to muscle-invasive diseases, of which the underlying key molecular events have yet to be addressed.

**Results:**

Here, we identified poly(A) binding protein nuclear 1 (PABPN1), a general factor of alternative polyadenylation (APA), was downregulated in BC. Overexpression and knockdown of PABPN1 significantly decreased and increased BC aggressiveness, respectively. Mechanistically, we provide evidence that the preference of PABPN1-bound polyadenylation signals (PASs) depends on the relative location between canonical and non-canonical PASs. PABPN1 shapes inputs converging on Wnt signaling, cell cycle, and lipid biosynthesis.

**Conclusions:**

Together, these findings provide insights into how PABPN1-mediated APA regulation contributes to BC progression, and suggest that pharmacological targeting PABPN1 might have therapeutic potential in patients with BC.

**Supplementary Information:**

The online version contains supplementary material available at 10.1186/s13578-023-00997-6.

## Background

Bladder cancer (BC) is one of the most common malignancies worldwide, with 400 000 new cases diagnosed and 165 000 cancer-related deaths every year [1]. Most of the patients with BC are presented with noninvasive disease at the time of diagnosis, while 10–20% of patients will progress to muscle-invasive bladder cancer (MIBC), which is usually more aggressive and has higher fatality rates [2]. In general, MIBC shows highly malignant features such as rapid recurrence and remarkable proliferative and metastatic potential [3]. However, the underlying mechanisms that drive the aggressiveness of BC remain elusive. Therefore, it is important to characterize key molecular events responsible for BC progression, so as to identify effective targets for precise treatment against BC.

Alternative polyadenylation (APA) has been identified as a crucial step during the post-transcriptional regulation of eukaryotic mRNAs [4]. APA is derived from the existence of multiple polyadenylation signals (PASs, AAUAAA or a similar sequence) within the same transcript, and normally results in the production of different mRNA isoforms with unchanged protein sequences [5]. Emerging evidence suggests that APA is critical for controlling mRNA stability, localization, translation, protein localization and coding [6]. APA is regulated by over 60 trans-regulators, of which ~ 20 are core proteins, including cleavage and polyadenylation specificity factors (CPSFs), cleavage stimulation factors (CSTFs), and cleavage factors I and II (CFIm and CFIIm) [7]. Recently, many advances in RNA sequencing facilitate interpretations of the complexity of APA events, thus providing new perspectives on stem cell differentiation, tissue development, and cancer biology.

The poly(A) binding protein nuclear 1 (PABPN1) is a ubiquitously expressed RNA binding protein that plays an important role in mRNA polyadenylation [8]. As a nuclear protein, PABPN1 functions by enhancing the processivity of poly (A) polymerase to regulate poly (A) tail length [9]. In addition, PABPN1 modulates APA by regulating 3’ untranslated region (3’UTR) length, which could influence downstream post-transcriptional regulatory mechanisms [10]. The PABPN1 gene is located at chromosome 14q11.2 and has been found to be linked to the pathogenesis of muscular dystrophies. Mutation of PABPN1 gene leads to oculopharyngeal muscular dystrophy (OPMD) and myotonic dystrophy [11]. Moreover, PABPN1 has been shown to contribute to the regulation of certain RNA processing events including mRNA splicing, and RNA turnover [12]. Using DNA double-strand breaks (DSB) repair pathway assays and mass spectrometry analysis, Michal et al. showed PABPN1 plays a role in regulating DSB repair, suggesting a novel ATM dependent-link between RNA binding proteins and the DNA damage response [13]. More recent studies have shown that PABPN1 is linked to human cancer progression [14, 15], but the role of PABPN1 in bladder cancer is unknown.

In this study, we identified PABPN1 as a tumor suppressor in BC. Enforced expression of PABPN1 inhibited, whereas silencing of PABPN1 stimulated the proliferative and metastatic potential of BC cells. Using DaPars algorithm based on RNA-sequencing (RNA-seq) [16, 17], we identified the APA-affected targets of PABPN1, and characterized the mechanism for PABPN1-mediated APA regulation. PABPN1 preferentially binds to proximal PASs in the 3’UTRs of target genes. By validating the cancer-related factors and pathways targeted by PABPN1, we determined the role of PABPN1 as a regulator of Wnt signaling, cell cycle, and lipid biosynthesis. Together, our study systematically identified an important role for PABPN1 and APA in BC progression.

## Results

### Upregulated PABPN1 may produce more long 3’UTR isoforms

**Fig. 1:**
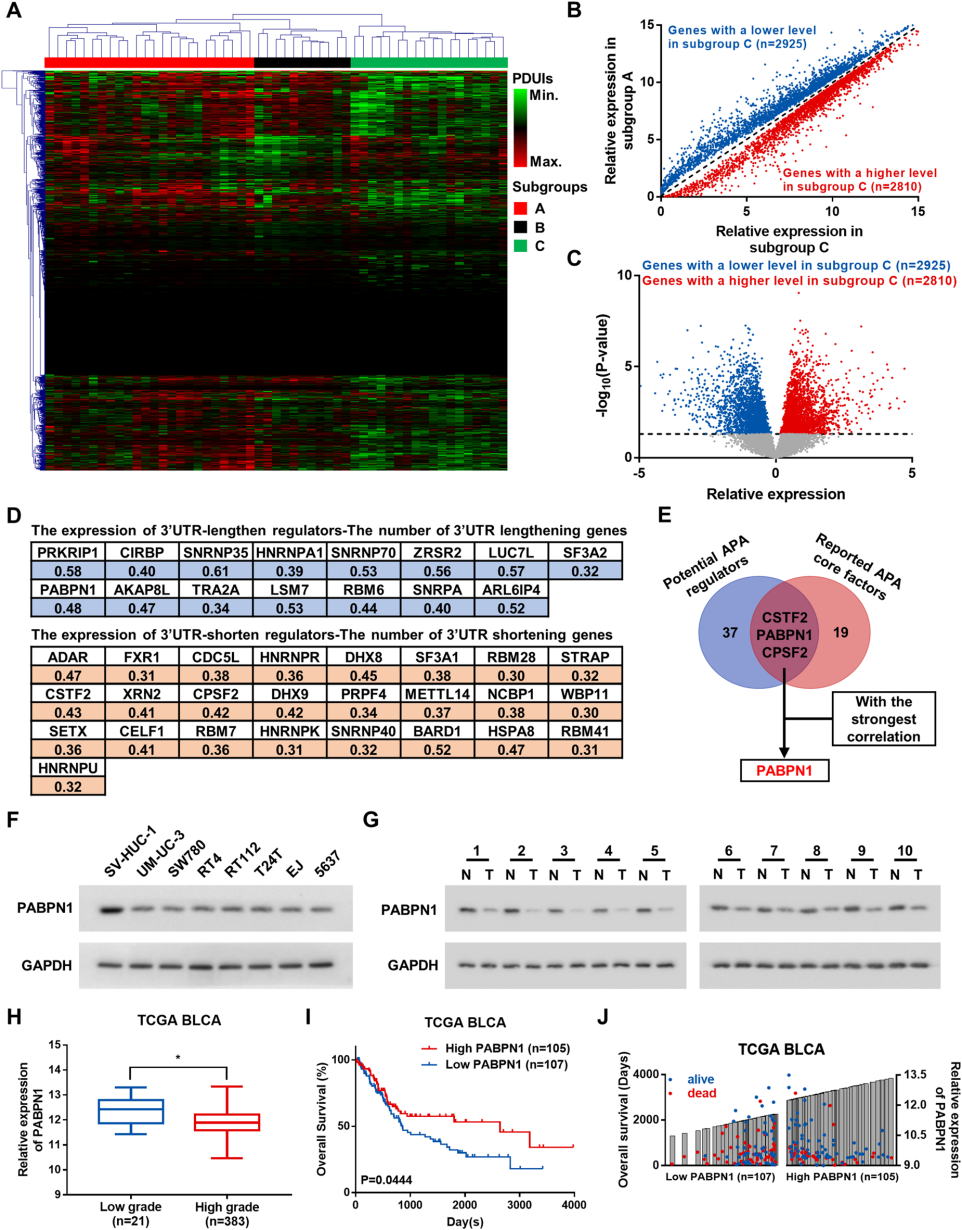
Potential APA regulator PABPN1 is downregulated in BC cells. **A** Hierarchical clustering of samples (columns) in TCGA BLCA based on PDUI scores of each gene (rows). **B** Scatter plot showing expression levels of DEGs in subgroup A and C. **C** Volcano plot showing relative expression of genes in subgroup C to subgroup A. **D** Correlation coefficients between the expression of 40 potential APA regulators and the number of genes undergoing 3’UTR lengthening or shortening in samples from TCGA BLCA dataset. **E** A venn diagram of potential APA regulators and reported APA core factors. **F** PABPN1 protein levels in human normal urothelial epithelial cell line and BC cell lines. GAPDH was used as a loading control. **G** PABPN1 protein levels in BC tissues (T) and adjacent non-tumorous tissues (N). GAPDH was used as a loading control. **H** Relative expression of PABPN1 in low/high grade BC samples from TCGA. **I** Overall survival in patients from TCGA BLCA with PABPN1 low versus high expression. **J** Overall survival time (left axis, the height of dots), survival state, and PABPN1 expression (right axis, the height of bars) in patients from TCGA BLCA

To screen out potential APA regulators in BC, we firstly clustered TCGA BLCA samples (columns) into 3 subgroups according to 3’UTR lengths (represented by PDUIs) of each gene (rows) (termed A, B, and C) (Fig. 1A). Considering the positive correlation between PDUI and 3’UTR length, samples in subgroup A generally contained longer 3’UTRs, while those in subgroup C displayed relatively shorter 3’UTRs, and the differentially expressed genes (DEGs) between these two subgroups may responsible for this difference. Then, we analyzed DEGs. Compared with subgroup A, 2925 genes presented significantly lower expression and 2810 genes showed significantly higher expression in subgroup C (Fig. 1B, C). To narrow it down, DEGs were intersected with several APA related genesets in Gene Ontology (GO), including mRNA processing (GO: 0006397), RNA binding (GO: 0003723), and Nucleus (GO: 0005634). In total, 41 downregulated genes (potential 3’UTR-lengthen regulators) and 53 upregulated genes (potential 3’UTR-shorten regulators) were identified (Additional file 1: Fig S1A, B, Additional file 4: Table S1). Among these 94 genes, many APA regulators, such as PABPC1, PABPN1, CPSF1, SF3B1 PCF11, SF3A1, NUDT21, CSTF2, CPSF2, and SF3B2, were identified. To further determine the key APA regulators in BC, we analyzed the correlation between the expression of these 94 potential regulators and the number of genes undergoing 3’UTR lengthening or shortening in samples from TCGA BLCA dataset, and further shortlisted 40 genes as potential APA regulators (r > 0.3, P < 0.05) (Fig. 1D). By overlapping with the reported APA core factors [18], 3 genes (CSTF2, PABPN1, and CPSF2) were screened out as key APA regulators in BC. Among them, the expression of PABPN1 had the strongest correlation (r = 0.48) with the number of genes undergoing 3’UTR alteration (Fig. 1E). These observations suggest that PABPN1 might be a key APA regulator in BC and play an important role in BC progression.

### PABPN1 is substantially decreased in BC and correlates with favorable prognosis

To investigate the potential roles of PABPN1 in BC, we first assessed PABPN1 expression in cell lines and 10 paired BC tissues using qRT-PCR and western blot, the results showed that the expression level of PABPN1 was markedly decreased in the seven tested BC cell lines compared with human normal urothelial epithelial cell line (SV-HUC-1) (Additional file 1: Fig S1C and F). In addition, PABPN1 was significantly downregulated in BC tissues (T) compared with matched adjacent noncancerous tissues (N) (Additional file 1: Fig S1D and G). Further comparative analysis using TCGA and the Gene Expression Omnibus (GEO) databases showed that the expression level of PABPN1 was significantly lower in BC patients with higher pathological grade (Fig. 1H and Additional file 1: Fig S1E), muscle invasive diseases (Additional file 1: Fig S1F), and lymph nodes metastasis (Additional file 1: Fig S1G). Furthermore, low expression of PABPN1 was statistically associated with poor clinical prognosis in BC patients (Fig. 1I, I). Collectively, these results reveal that robust decrease of PABPN1 may play an important role in the progression of BC.

### Influence of expression levels of PABPN1 on tumorigenicity and metastasis of BC in vitro and in vivo

**Fig. 2:**
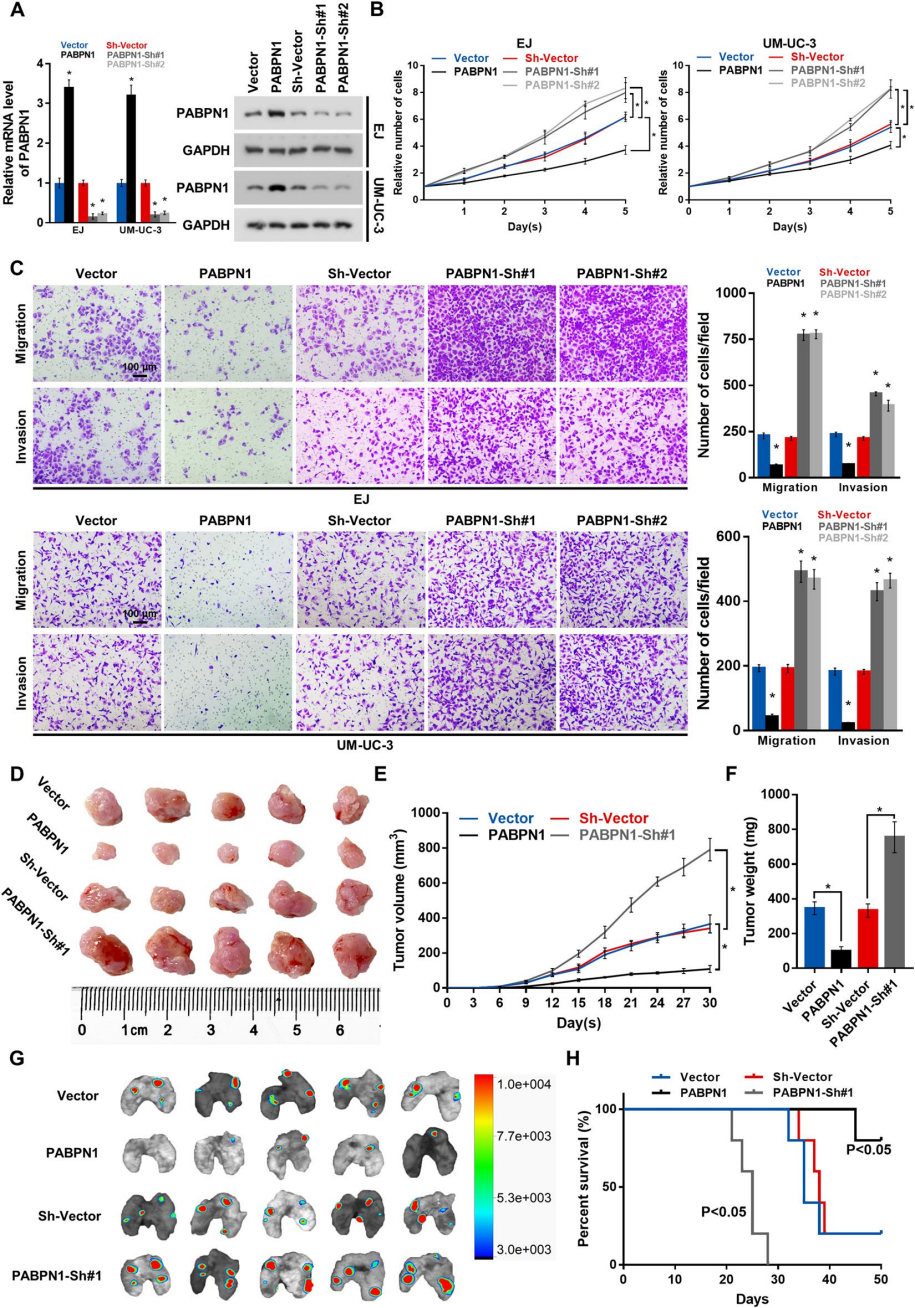
PABPN1 inhibits the progression of BC cells. **A** The qRT-PCR and western blot analysis of PABPN1 levels in BC cells stably overexpressed, or silenced for PABPN1. **B** Proliferation abilities of BC cells determined using the CCK-8 assay. **C** Representative pictures of migrating and invading cells analyzed by Transwell assay. 200 × . **D** Xenograft tumors of nude mice from indicated groups. **E** Tumor volumes measured every third day. **F** Tumor weight of all mice in each group. **G** Pulmonary metastasis of BC cells detected using In Vivo Optical Imaging System. **H** Survival curves for nude mice in the tumor metastasis assay. Data are presented as the mean ± SD of three independent experiments. * P < 0.05

To further explore the function of PABPN1 in BC progression, we created stable PABPN1-overexpressing (PABPN1) and -knockdown (PABPN1-Sh#1 and PABPN1-Sh#2) cell lines using EJ and UM-UC-3 BC cells (Fig. 2A). As shown in Fig. 2B, overexpression of PABPN1 inhibited, while silencing of PABPN1 expression promoted, cell proliferation in both BC cell lines. Transwell assays showed that PABPN1 upregulation markedly decreased, while knockdown of PABPN1 caused an apparent increase in cell migration and invasion (Fig. 2C). In vivo assays showed that overexpression of PABPN1 in BC cells significantly reduced tumor volume and weight of the subcutaneous xenografts, while knockdown of PABPN1 increased the tumor-initiating ability of BC cells (Fig. 2D–F). Moreover, overexpression of PABPN1 decreased the number of metastases in the lungs and extended the survival of mice, whereas knockdown of PABPN1 showed the opposite results (Fig. 2G, H). Together, these results suggest that PABPN1 inhibits BC cells proliferation and metastasis both in vitro and in vivo.

### PABPN1 modulates mRNA alternative polyadenylation in BC cells

**Fig. 3:**
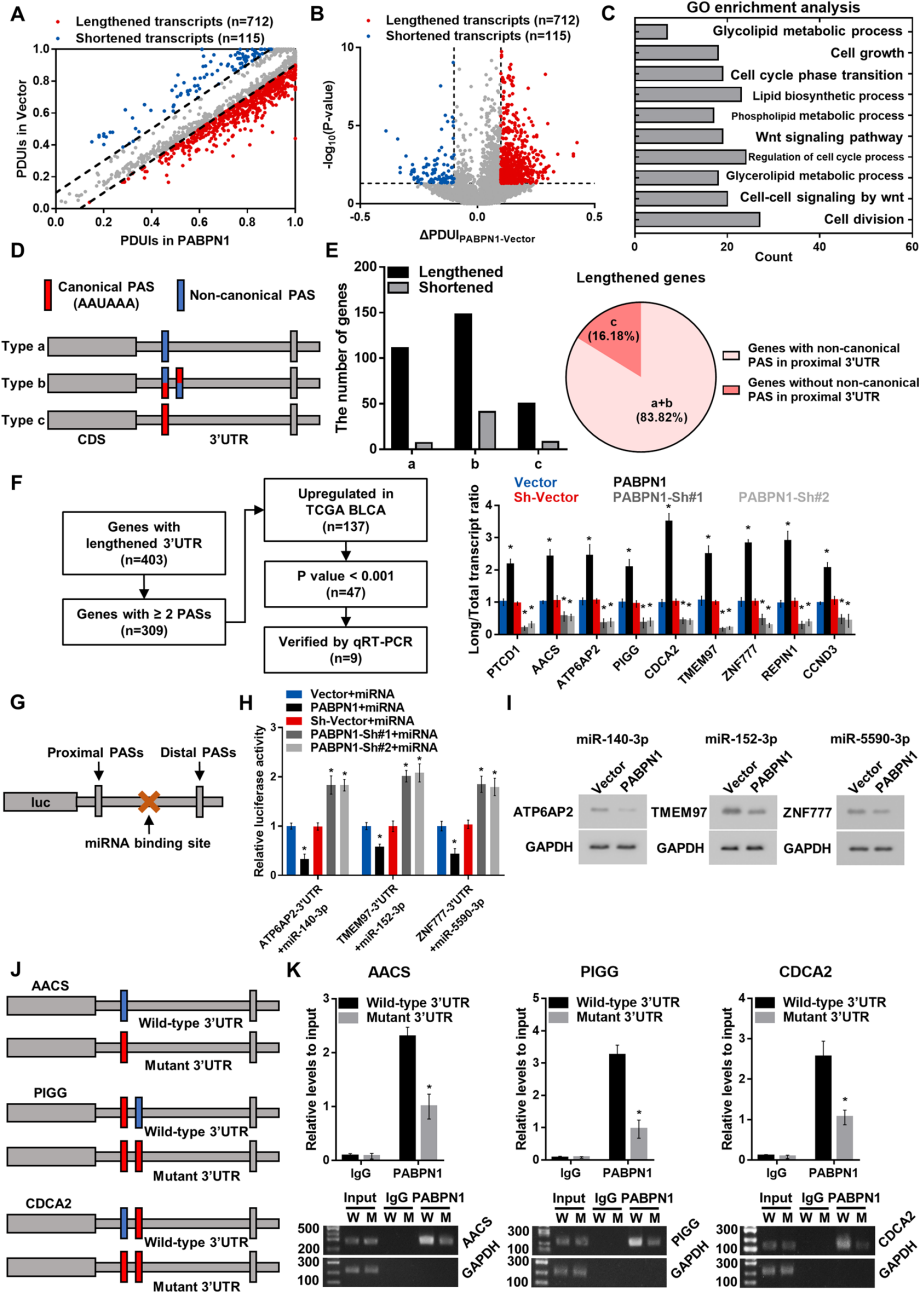
Upregulated PABPN1 leads to more transcripts with longer 3’UTRs in BC cells. **A** Scatter plot showing PDUIs of transcripts with significant (P < 0.05) lengthened (n = 712) or shortened (n = 115) 3’UTRs in PABPN1 overexpressing and control BC cells. **B** Volcano plot showing ΔPDUI_PABPN1-Vector_ of transcripts generated by DaPars algorithm. ΔPDUI_PABPN1-Vector_ = MeanPDUI_PABPN1_–MeanPDUI_Vector_. **C** GO enrichment analysis of PABPN1 target genes with lengthened 3’UTRs. **D** Schematic illustration showing the PASs distribution of type a-c of PABPN1 target genes. **E** The number of genes in type a-c with lengthened or shortened 3’UTRs (left), the proportion of 3’UTR lengthened genes with or without non-canonical PAS in proximal 3’UTRs (right). **F** The flow chart for screening out potential targets of PABPN1 (left), the long transcript-to-total transcript ratio of potential target genes upon PABPN1 overexpression or knockdown measured by qRT-PCR (right). **G** Diagram showing luciferase reporters containing miRNA binding sites located between proximal and distal PASs in 3’UTR. **H** Dual-luciferase reporter assay revealing the PABPN1-mediated elongation effect on 3’UTR. **I** Western blot assay revealing the PABPN1-mediated gene silencing effect of miRNA. **J** Diagram showing vectors containing wild-type or mutant 3’UTRs of PABPN1 target genes. **K** Binding capacities of PABPN1 to wild-type or mutant 3’UTRs evaluated by RIP assays with anti-PABPN1 antibody. IgG was used as a negative control. Data are presented as the mean ± SD of three independent experiments. * P < 0.05

To systematically identify the target genes of PABPN1, we performed DaPars algorithm based on RNA-seq to determine the global APA profile of BC cells in response to PABPN1 overexpression. As shown in Fig. 3A, B, 712 transcripts with longer 3’UTRs were generated in response to PABPN1 overexpression, while only 115 transcripts showed significant shortening. Moreover, GO enrichment analysis revealed that the potential PABPN1 target genes were enriched in important pathways including cell growth, cell cycle, Wnt signaling, and lipid biosynthetic process (Fig. 3C). Notably, a similar predicted enrichment analysis in our previously published dataset also identified Wnt signaling pathway genes as main downstream targets of NUDT21, supporting a link between APA and BC pathogenesis [19]. Cumulatively, these data support that PABPN1 is a key regulator of BC-promoting pathways through its APA regulation activity.

### PABPN1 regulation of proximal PAS in BC

Given that PABPN1 is more likely to recognize and suppress the usage of weak proximal and non-canonical PASs in oculopharyngeal muscular dystrophy [10], we assessed whether PABPN1 diminishes the proximal PASs usage in cancer cells. Based on the DaPars analysis upon PABPN1 overexpression, we identified rather more target genes with lengthened 3’UTRs, indicating that PABPN1 is more likely to bind to proximal PAS regions in BC cells (Fig. 3A, B, E, left panel). Then we divided PABPN1 target genes into three types based on the distributions of canonical and non-canonical PASs in 3’UTRs: Type a, the proximal region contains only non-canonical but not canonical PAS. Type b, the proximal region contains both canonical and non-canonical PASs. Type c, the proximal region contains only canonical but not non-canonical PAS (Fig. 3D). Notably, most (83.82%) of potential PABPN1 targets harbor non-canonical PASs in proximal 3’UTR, suggesting that PABPN1 prones to bind to non-canoncial PASs in proximal 3’UTR (Fig. 3E, right panel).

To test our conjectures put forward above, we further screened potential targets of PABPN1 by the following criteria: (1) with at least 2 PASs in 3’UTR; (2) upregulated in BC; (3) undergoing significant lengthening in 3’UTRs upon PABPN1 overexpression (P < 0.001) (Fig. 3F, left panel). A total of 47 genes were filtered out. Subsequently, the long transcript-to-total transcript ratio of these 47 genes upon PABPN1 overexpression or knockdown were measured and calculated by qRT-PCR (Fig. 3F, right panel, and Additional file 2: Figure S2A). Eventually, 9 genes (PTCD1, AACS, ATP6AP2, PIGG, CDCA2, TMEM97, ZNF777, REPIN1, and CCND3) were selected as PABPN1 targets. 3’UTR profiles of these genes were displayed in Additional file 2: Fig S2B.

Next, dual-luciferase reporters containing 3’UTR of ATP6AP2 (Type a), TMEM97 (type b), and ZNF777 (Type c) were constructed and transfected into PABPN1-overexpressing BC cells. Then miRNAs, whose binding sites are located between proximal and distal PASs in 3’UTR of ATP6AP2 (miR-140-3p), TMEM97 (miR-152-3p), and ZNF777 (miR-5590-3p) respectively, were used to examine the influence of PABPN1 on 3’UTR length (Fig. 3G). The results showed that overexpressing PABPN1 could augment the binding of miRNAs by elongating 3’UTRs (Fig. 3H), and raise efficiency of miRNA-mediated gene silencing (Fig. 3I), further implying the preference of PABPN1 on proximal PAS. Moreover, we constructed vectors expressing PABPN1 target genes with wild-type or mutant (for genes in type a and type b, the proximal non-canonical PAS mutated into the canonical PAS; for genes in type c, the proximal canonical PAS mutated into the non-canonical PAS) 3’UTRs (Fig. 3J and Additional file 2: Fig S2C), and performed the RIP assay with anti-PABPN1 antibody to evaluate the binding capacity of PABPN1 to non-canonical and canonical PASs in proximal region of 3’UTR. Results showed that PABPN1 was more likely to bind to proximal non-canonical PAS in BC cells (Fig. 3K and Additional file 2: Fig S2D). These results strongly suggest that when there is non-canonical PAS in the proximal region of 3’UTR, it is favored even if the most proximal PAS is canonical. Otherwise, when the proximal 3’UTR contains only canonical PAS, PABPN1 binds priorly to the proximal canonical PAS.

### PABPN1-mediated APA drives the expression of BC-promoting genes

**Fig. 4:**
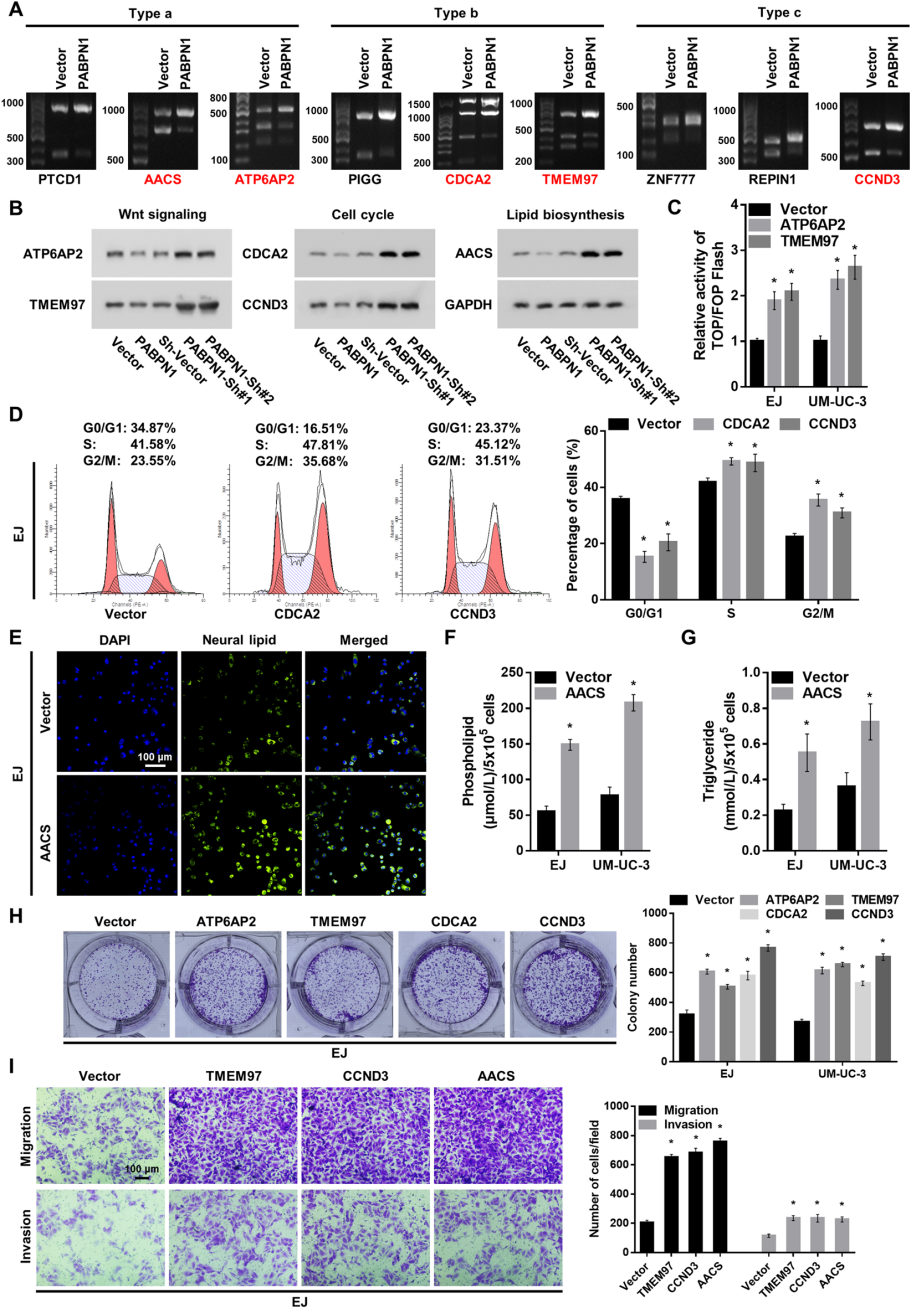
PABPN1 regulates the 3’UTR lengths of ATP6AP2, TMEM97, CDCA2, CCND3, and AACS. **A** Abundance of transcripts with different 3’UTR lengths identified by 3’RACE assay. **B** Western blot assay measuring levels of PABPN1 targeted, Wnt signaling (ATP6AP2 and TMEM97), cell cycle (CDCA2 and CCND3), and lipid biosynthesis (AACS) related genes. GAPDH was used as a loading control. **C** Analysis of TOP/FOP luciferase reporter activity in cells stably transfected with ATP6AP2 or TMEM97 overexpressing vectors. **D** Flow cytometric analysis showing the cell cycle distribution of cells stably transfected with CDCA2 or CCND3 overexpressing vectors. **E** The neutral lipids content in cells stably transfected with AACS overexpressing vector. Neutral lipids were stained with BODIPY 493/503 dye (green). Nuclei were stained with Hoechst (blue). 200 × . **F** Quantification of phospholipid levels in AACS-overexpressing cells. **G** Quantification of triglyceride levels in AACS-overexpressing cells. **H** Proliferation of BC cells shown by colony formation assay. **I** Representative pictures of migrating and invading cells analyzed by Transwell assay. 200 × . Data are presented as the mean ± SD of three independent experiments. * P < 0.05

We next validated representative APA-affected genes reflecting the identified pathways. We focused on target genes that (i) are involved in cell proliferation, including Wnt and cell cycle signalings, (ii) have previously been associated with BC pathogenesis or (iii) play a pivotal role in other pathways (eg, lipid biosynthesis) globally involved in tumorigenesis. Based on the criteria, we selected a panel of PABPN1 target genes, including ATP6AP2, TMEM97, CDCA2 CCND3, and AACS, all of which displayed more transcripts with longer 3’UTRs (Fig. 4A), and exhibited decreased protein expression with increased PABPN1 levels (Fig. 4B). These changes are likely functional as we also found the expected downstream alterations of the Wnt signaling (Fig. 4C), cell cycle (Fig. 4D and Additional file 3: Figure S3A), and lipid biosynthesis (Fig. 4E, G, Additional file 3: Fig S3B). Moreover, upregulation of ATP6AP2, TMEM97, CDCA2, and CCND3 led to a significant increase in the proliferation in BC cells (Fig. 4H and Additional file 3: Fig S3C), overexpression of TMEM97, CCND3, and AACS resulted in significantly increased migration and invasion abilities of BC cells (Fig. 4I and Additional file 3: Fig S3D). These results indicates that PABPN1 target genes promote BC progression.

### PABPN1 regulates Wnt signaling activation, cell cycle progression, and lipid biosynthesis in BC

**Fig. 5:**
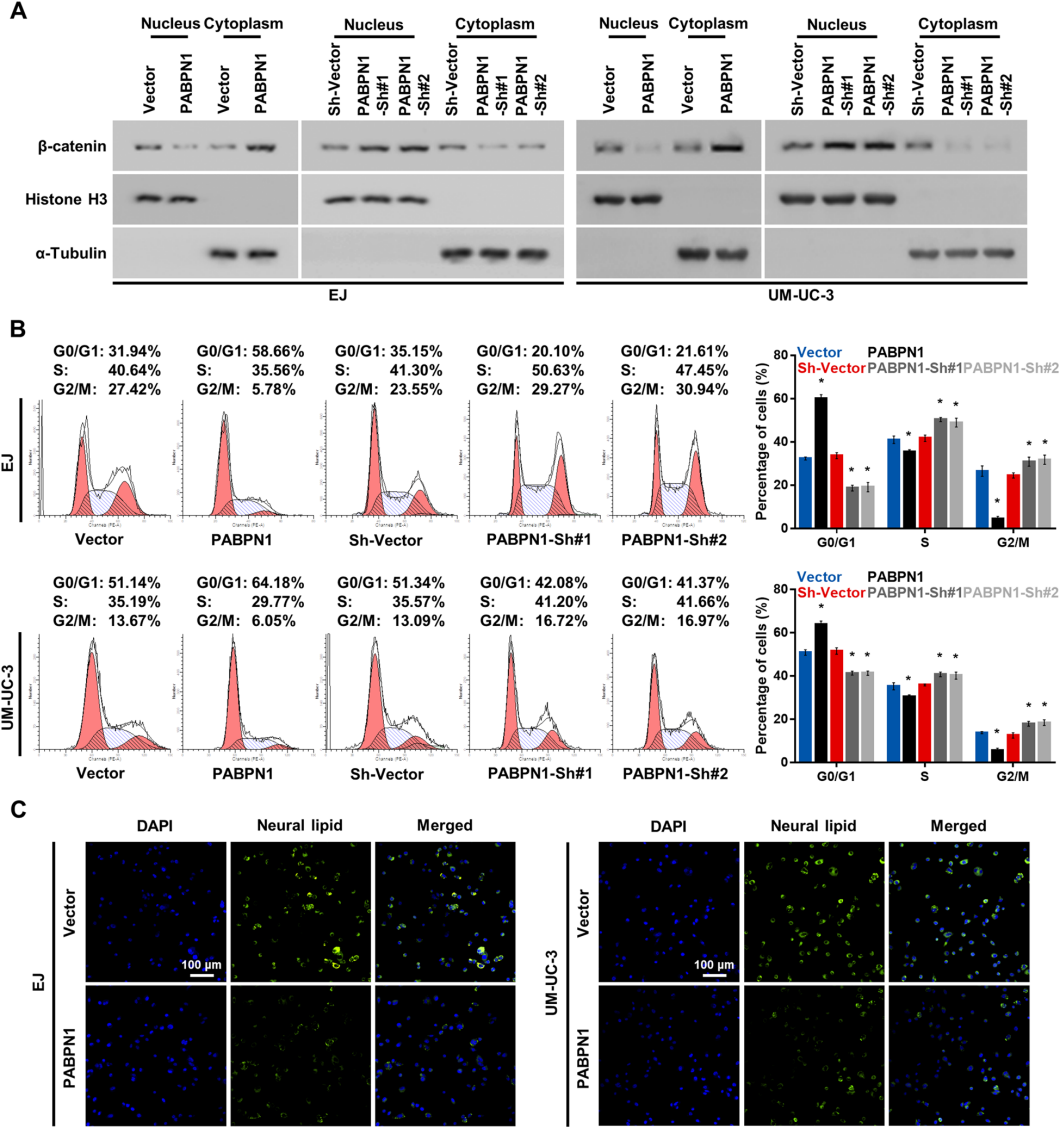
PABPN1 suppresses Wnt signaling, cell cycle progression, and lipid biosynthesis of BC cells. **A** Western blot analysis of β-catenin expression in nucleus and cytoplasm of BC cells. α-Tubulin and Histone H3 were used as loading controls for cytoplasmic and nuclear fractions, respectively. **B** Cell cycle distribution of indicated BC cells analyzed by flow cytometry. **C** The neutral lipids content in indicated cells detected with BODIPY 493/503 dye (green). Nuclei were stained with Hoechst (blue). 200 × . Data are presented as the mean ± SD of three independent experiments. * P < 0.05

To gain an insight into the mechanisms mediating the role of PABPN1 in tumor growth and metastasis, western blot assays were performed, which showed that overexpression of PABPN1 significantly inhibited, whereas silencing of PABPN1 enhanced, Wnt signaling activity (Fig. 5A). Flow cytometry assays revealed the inhibiting effect of PABPN1 on BC cells (Fig. 5B). Furthermore, the level of neural lipid was remarkably decreased in PABPN1-overexpressing cells (Fig. 5C). Taken together, these results further manifested the negative correlation of PABPN1 level with Wnt signaling pathway activation, cell cycle, and lipid biosynthesis.

### Clinical association of PABPN1 expression with Wnt signaling pathway, cell cycle, and lipid biosynthesis in human BC.

**Fig. 6:**
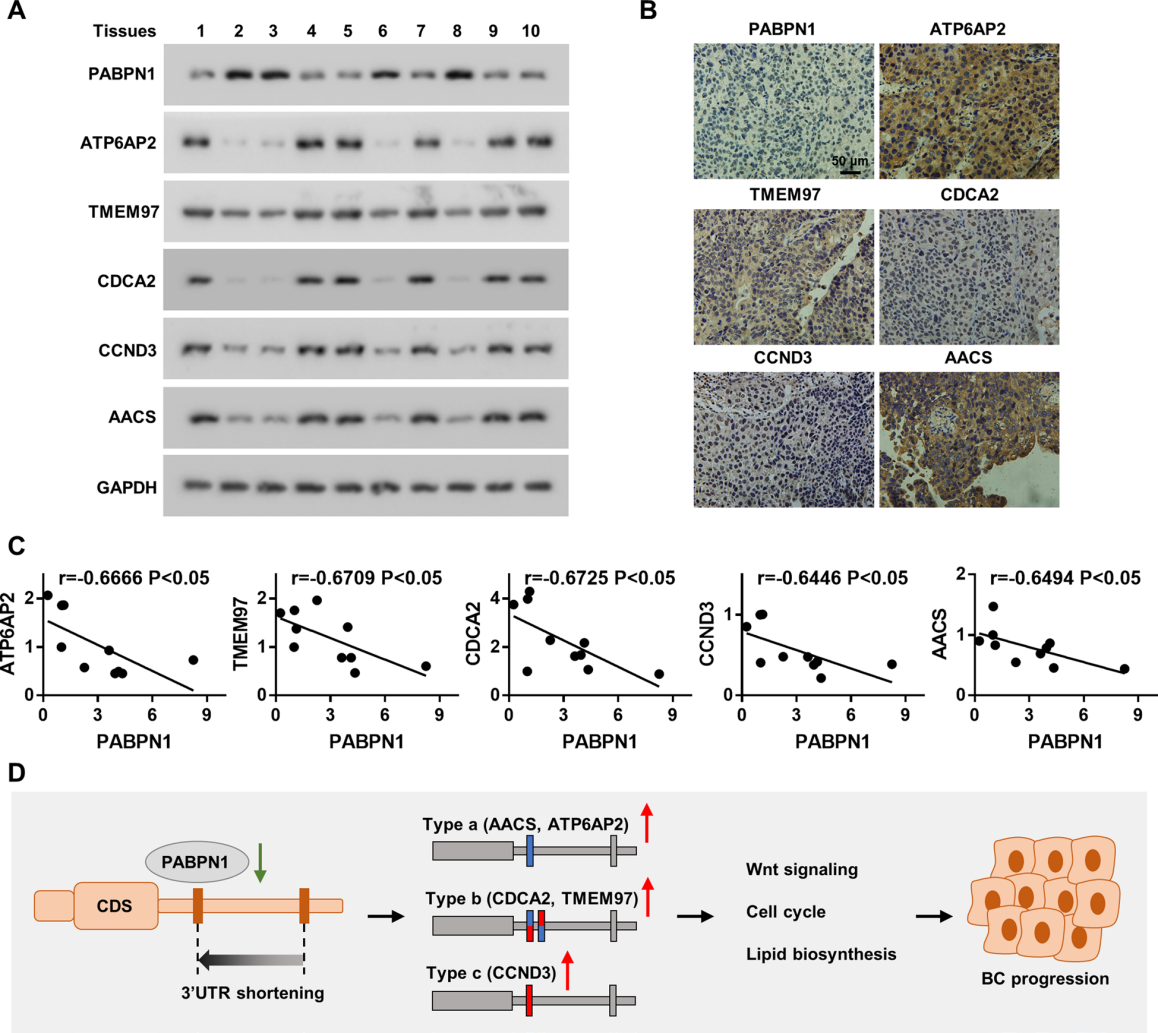
Clinical relevance between PABPN1 and its targets. **A** Western blot assay showing protein levels of PABPN1, ATP6AP2, TMEM97, CDCA2, CCND3, and AACS in clinical BC tissues. GAPDH was used as a loading control. **B** Representative IHC images of PABPN1, ATP6AP2, TMEM97, CDCA2, CCND3, and AACS expression in BC specimens. 400 × . **C** Correlation analysis between PABPN1 and ATP6AP2, TMEM97, CDCA2, CCND3, or AACS levels. **D** Schematic illustration showing the regulation mechanism of PABPN1 in BC

We further examined the correlation of PABPN1 and Wnt signaling pathway, cell cycle, and lipid biosynthesis in clinical tissue samples. The expression of PABPN1 correlated negatively with the levels of ATP6AP2, TMEM97, CDCA2, CCND3, and AACS (Fig. 6A–C). Collectively, our results indicate that the downregulation of PABPN1 in BC activates the Wnt signaling pathway, and promotes cell cycle progression and lipid biosynthesis, which consequently promotes the aggressiveness of BC (Fig. 6D).

## Discussion

The functions of PABPN1 through modulating APA patterns have been investigated in human cancers. However, it is not clear whether PABPN1 play a role in regulating gene expression and function in BC. In this study, we demonstrated for the first time that PABPN1 functions as a tumor suppressor in BC. Decreased expression of PABPN1 results in 3’UTRs shortening of a subset of target genes, some of which are involved in Wnt signaling, cell cycle, and lipid biosynthesis. Collectively, our findings shed light on the molecular mechanisms underlying the effect of PABPN1 on the modulation of BC progression.

Based on our data, we classify the PABPN1 targets into three main types. For genes whose proximal 3’UTR contain only non-canonical but not canonical PAS (Type a), PABPN1 prefers to bind to the proximal non-canonical PAS, and cleavage/polyadenylation occurs at the distal PAS. For genes that have both canonical and non-canonical PAS in the proximal 3’UTR (Type b), PABPN1 tends to bind to the non-canonical PAS, even when the most proximal PAS is canonical. Type a and b are the most common type of PABPN1 targets occurring in more than 80% of target genes. For genes that have only canonical but not non-canonical PAS in the proximal 3’UTR (Type c), PABPN1 is more prone to bind to proximal canonical PAS. Our findings indicate that, when there is non-canonical PAS in the proximal region of 3’UTR, it is favored even if the most proximal PAS is canonical. In another situation, when the proximal 3’UTR contain only canonical PAS, PABPN1 preferentially binds to the proximal canonical PAS, leading to an increase in distal PAS utilization, and therefore producing longer mRNA. Previous study showed that PABPN1 suppresses the usage of weak proximal and non-canonical PAS [10], while there is lack of studies in elucidating the mechanism of how PABPN1 interact with canonical PAS regions. Our model extends the classical model for PABPN1’s role in suppressing the usage of proximal PAS by incorporating the relative location between canonical and non-canonical PASs as a new parameter, and explains how PABPN1 can differentially interact with PAS regions in different transcripts.

Several signaling pathways, including Wnt, NF-κB, PI3K/Akt, and TGF-β have been shown to regulate cell proliferation and progression of BC [20, 21]. To examine the role of PABPN1 in pathway regulation, we performed a functional annotations assay and found that PABPN1 targets were involved in Wnt and cell cycle pathways. We verified that ATP6AP2 and TMEM97, two genes involved in the Wnt signaling, had 3’UTR shortening and elevated protein expression upon PABPN1 knockdown. Additionally, we verified that PABPN1 could regulate the 3’UTR length of CCND3 and CDCA2, which have been found to be involved in cyclin pathway and cell cycle regulation. Importantly, amplification of either gene in BC is correlated with tumor aggressiveness and progression [22, 23]. Target genes with shortened 3’UTRs can be more stable as miRNAs and RNA-binding proteins originally bind to the longer form are released, thus resulting in the enhanced protein translation [24]. Consistent with this findings, PABPN1 depletion promotes BC cell proliferation and DNA replication. Therefore, our data suggest that Wnt and cell cycle pathways are key downstream targets of PABPN1 in BC. In addition, as our previous study showed that NUDT21 inhibits bladder cancer growth and metastasis via suppressing the expression of Wnt signaling targets [19], we compared the genes whose APA is regulated by PABPN1 in the present study and those by NUDT21, and found few overlap target genes. Thus, although the directionalities of APA regulation by PABPN1 and NUDT21 are the same, they seem to regulate different sets of target genes in BC.

While we found a subset of Wnt signaling and cell cycle-related genes as potential targets of PABPN1, other genes were also found to be regulated by PABPN1. Indeed, we found more long isoforms of lipid biosynthesis-related genes AACS in PABPN1-overexpressed cells. Moreover, knockdown of PABPN1 enhanced protein expression of AACS and activated lipid biosynthetic signaling. As emerging evidence indicates that lipid metabolism plays a central role in the development and pathogenesis of BC [25, 26], our findings reveal a potential positive contribution of targeting lipid metabolism to the treatment of BC.

## Conclusions

In conclusion, our study identifies PABPN1 as a tumor suppressor in BC. Moreover, PABPN1 downregulation was sufficient to promote BC cell proliferation, cell cycle progression, migration and invasion via regulation of APA of target genes. Additionally, this work unravels an earlier undefined mechanism of how PABPN1 differentially interact with PAS regions in different transcripts. Taken together, our findings suggest that PABPN1 might be a promising therapeutic target for BC, although more clinical research is needed.

## Methods

### Cell culture

The uroepithelial cell line SV-HUC-1 (RRID: CVCL_3798) and bladder cancer cell lines UM-UC-3 (RRID: CVCL_1783), SW780 (RRID: CVCL_1728), RT4 (RRID: CVCL_0036), and 5637 (RRID: CVCL_0126) were purchased from the Cell Bank of Chinese Academy of Sciences (Shanghai, China), RT112 (RRID: CVCL_1670) was purchased from DSMZ, T24T (RRID: CVCL_M892) and EJ (RRID: CVCL_2893) were gifted by Dr. Guosong Jiang of Wuhan Union Hospital. All cell lines were cultured in appropriate complete medium and maintained at 37 °C in 5% CO_2_ condition.

All cell lines have been authenticated using short tandem repeat profiling within the last 3 years and tested for mycoplasma contamination by DAPI staining. All experiments were performed with mycoplasma-free cells.

### Clinical tissue specimens

Fresh tumor specimens and adjacent non-tumorous tissues of BC patients were obtained from Wuhan Union Hospital. None of patients involved in this study received preoperative radiotherapy or chemotherapy. All procedures related to clinical specimens were approved by the ethics committee of the Union Hospital affiliated to Huazhong University of Science and Technology and undertaken in accordance with guidelines set forth by the Declaration of Helsinki.

### Quantitative real-time PCR (qRT-PCR)

Total RNA that extracted from cell lines or fresh tissues with TRIzol reagent (Invitrogen, CA, USA) was reverse transcribed into cDNA using PrimeScript RT Reagent Kit (Takara, Kyoto, Japan). To quantify the expression level, qRT-PCR was performed on a StepOnePlus Real-Time PCR System (Applied Biosystems, CA, USA). 2^–ΔΔCT^ method was used to calculated the relative expression. GAPDH was used as the internal control. Primers used in this study were synthesized by Sangon Biotech (Shanghai, China) (Additional file 5: Table S2).

### Western blot

Cellular or tissue protein was extracted with RIPA lysis buffer (Servicebio, Wuhan, China), and western blot was performed as previously described [27]. Antibodies use for western blot: anti-PABPN1 (66807-1-Ig, Proteintech, USA), anti-GAPDH (60004-1-Ig, Proteintech, USA), anti-ATP6AP2 (10926-1-AP, Proteintech, USA), anti-TMEM97 (26444-1-AP, Proteintech, USA), anti-ZNF777 (NBP1-03344, Novus Biologicals, USA), anti-CDCA2 (17701-1-AP, Proteintech, USA), anti-CCND3 (26755-1-AP, Proteintech, USA), anti-AACS (ab237802, Abcam, UK), anti-β-catenin (51067-2-AP, Proteintech, USA), anti-Histone H3 (17168-1-AP, Proteintech, USA), and anti-α-Tubulin (66031-1-Ig, Proteintech, USA).

### Lentivirus construction and infection

Human PABPN1 cDNA was amplified and cloned into GV705 lentivirus vector (CMV enhancer-MCS-3FLAG-sv40-puromycin) (GeneChem, Shanghai, China). Oligos of PABPN1 shRNAs were synthesized and inserted into GV112 vector (hU6-MCS-CMV-Puromycin) (GeneChem, Shanghai, China). To establish stable cell lines, BC cells were infected with lentivirus vectors expressing PABPN1 (PABPN1) or PABPN1-shRNAs (PABPN1-Sh#1 and PABPN1-Sh#2), and were selected with 2 μg/ml puromycin for at least 30 days.

### Cell counting kit-8 (CCK-8) assay

For CCK-8 assay, cells from different groups were seeded into a 96-well plate at 3 × 10^3^ cells per well and incubated at 37 °C. To detect the proliferation ability, cells were incubated in 100 μL of complete medium adding with 10 μL of CCK-8 reagent (DOJINDO, Kumamoto, Japan) for 2 h at 37 °C. The absorbance, which represented the number of surviving cells, was measured at 450 nm using a microtiter plate reader.

### Cell migration and invasion assays

Transwell system (Corning, NY, USA) was used to evaluated the migration or invasion capacity of BC cells. In brief, cells suspended in serum-free medium were seeded in upper chambers without (for migration assay) or with (for invasion assay) Matrigel, while the serum-containing medium was added into lower chambers. After 24 h, cells remaining on the top surface were gently removed, and cells migrated or invaded to the lower surface were fixed, stained, and photographed, under a light microscope.

### Colony formation assay

BC cells were seeded in 6-well plates at the density of 500 cells per well, and incubated at 37 °C in 5% CO_2_ for 2 weeks. Cell colonies were immobilized, dyed and recorded using the digital single-lens reflex camera (D610, Nikon, Japan).

### Cell cycle analysis

For cell cycle analysis, BC cells were collected and fixed in 70% ethanol at 4 °C. After 0.5 h, cells were washed with PBS and incubated with PI/RNase Staining Buffer (BD Biosciences, CA, USA) in the dark for 30 min. Cell cycle was analyzed by BD LSRFortessa X-20 (BD Biosciences, CA, USA).

### Detection of neutral lipid, triglyceride, and phospholipid

Neutral lipid accumulation in BC cells was monitored by lipophilic fluorescence dye BODIPY 493/503 (Invitrogen, CA, USA). Briefly, cells were washed in Phosphate Buffered Saline (PBS), fixed with 4% paraformaldehyde, and stained with BODIPY 493/503 (1ug/mL) for 45 min at room temperature. Nuclei were counterstained with Hoechst (10 μg/mL) for 15 min. All fluorescence images were captured using a Nikon A1R-si Laser Scanning Confocal Microscope (Nikon, Tokyo, Japan). For quantitative estimation of phospholipid and triglyceride, the EnzyChrom Phospholipid Assay Kit and EnzyChrom Triglyceride Assay Kit (BioAssay Systems, CA, USA) were used according to manufacturer’s instructions.

### In vivo tumor growth and metastasis assays

Animal experiments involved in this study were conducted in the light of the Guide for the Care and Use of Laboratory Animals [28], and approved by the ethics committee of Tongji Medical College of the Huazhong University of Science and Technology. BALB/c-nu mice (male, 3–5 weeks of age) were randomly divided into 4 groups. For the tumor growth assay, BC cells were injected subcutaneously into the right axilla of each mouse. After injection, tumor volumes were observed using an external caliper and recorded using the equation (L × W^2^)/2. On day 30, all of the mice were euthanized, and tumors were excised, weighed, and photographed. For the metastasis assay, cells were injected into the tail-vein of each mouse. On day 50, all animals were euthanized by cervical dislocation, and lungs were excised and imaged with the In Vivo Optical Imaging System (In Vivo FX PRO, Bruker, MA, USA).

### Dual-luciferase reporter assay

Dual-luciferase reporters based on psiCHECK-2 vectors containing 3’UTRs of ATP6AP2, TMEM97, and ZNF777 were constructed, and then transfected into BC cells with corresponding miRNA mimics. After 48 h, the luciferase activities were measured with the Dual-Luciferase Reporter Assay System (Promega, WI, USA).

### Rapid amplification of cDNA 3’ ends (3’RACE)

Total RNA was extracted and reversely transcribed into cDNA using the primer 5ʹ-CCAGTGAGCAGAGTGACGAGGACTCGAGCTCAAGCTTTTTTTTTTTTTTTTT-3ʹ. Forward primers designed specifically for PTCD1, AACS, ATP6AP2, PIGG, CDCA2, TMEM97, ZNF777, REPIN1, and CCND3 were designed (Additional file 5: Table S2) and mixed with the reverse primer 5ʹ-CCAGTGAGCAGAGTGACG-3ʹ to amplify the cDNA 3’ ends. Then products were electrophoresed on agarose gels and recorded under the ultraviolet light.

### RNA binding protein immunoprecipitation (RIP) assay

For RIP assay, approximately 1 × 10^7^ BC cells transfected with AACS, PIGG, and CDCA2 overexpressing vectors harboring wild-type or mutant 3’UTR were collected, and lysed in polysome lysis buffer (100 mM KCl, 5 mM MgCl_2_, 10 mM HEPES–NaOH pH 7.0, 0.5% Nonidet P-40 supplemented with 1 mM dithiothreitol, 200 units/ml RNase OUT, and EDTA-free Protease Inhibitor Cocktail) on ice for 10 min. After the preparation of Protein A/G Magnetic Beads (MedChemExpress, NJ, USA) and immobilization of antibodies (anti-PABPN1, 66807-1-Ig, mouse anti-IgG1, 66360-1-Ig, Proteintech, USA), cell lysate was incubated with antibody-bead reaction overnight at 4 ℃. The next day, beads were washed with ice-cold NT-2 (50 mM Tris–HCl pH 7.4, 150 mM NaCl, 1 mM MgCl_2_, 0.05% Nonidet P-40) six times, and incubated with Proteinase K digestion buffer (1 × NT-2 buffer supplemented with 1% sodium dodecyl sulfate, 1.2 mg/ml Proteinase K) at 55 °C for 30 min. RNA Purification was conducted and binding products were detected using qRT-PCR.

### Heatmap analysis

We downloaded the Percentage of Distal polyA site Usage Index (PDUI) scores of every gene in The Cancer Genome Atlas Bladder Urothelial Carcinoma (TCGA-BLCA) samples from TC3A database (http://tc3a.org/) [29]. Genes favoring distal PAS usage (long 3’UTRs) have PDUI scores near 1, whereas genes favoring proximal PAS usage (short 3’UTRs) have PDUI scores near 0. Then, heatmap analysis and hierarchical clustering were conducted using MeV 4.9.0 application based on PDUI scores of genes in tumor samples. Three distinct subgroups were identified being characterized by different 3’UTR alteration patterns.

### RNA sequencing (RNA-Seq)

RNA-seq was performed with the help of HaploX (Jiangxi, China). Total RNA from EJ cells with or without PABPN1 overexpressing was extracted, and RNA purity (OD260/280 and OD260/230), concentration (ng/μL), and integrity (RIN) was determined. Then, the mRNA library was constructed, and the sequencing was performed on an Illumina PE150 platform.

### Dynamic analysis of alternative polyadenylation from RNA-Seq (DaPars)

To systematically investigate the impact of PABPN1 overexpressing on global APA landscape in BC cells, we utilized DaPars [16, 17], a well-established computational algorithm, to analysis the RNA-Seq files of PABPN1 overexpressing BC cell and the corresponding control cell. Briefly, DaPars uses a linear regression model to predict the proximal APA site and estimates the abundance of long form and short form of 3’ UTRs, then calculates the PDUI.

### Statistical analyses

Statistical analyses were performed using the GraphPad Prism 7 (GraphPad, CA, USA). Data were presented as mean ± SD and from at least three independent experiments. Pearson correlation coefficient was used to analyze the expression correlation. Survival curves were calculated according to the Kaplan–Meier method. Differences between groups were analyzed by Student’s t test and p-value < 0.05 was considered statistically significant.

## Supplementary Information

**Additional file 1: Figure S1**. Potential APA regulator PABPN1 is downregulated in BC cells. (A) Venn diagrams showing 41 potential 3’UTR-lengthen regulators in BC. (B) Venn diagrams showing 53 potential 3’UTR-shorten regulators in BC. (C) PABPN1 mRNA levels in human normal urothelial epithelial cell line and BC cell lines. (D) PABPN1 mRNA levels in BC tissues (T) and adjacent non-tumorous tissues (N). (E) Relative expression of PABPN1 in low/high grade BC samples from GSE13507. (F) Relative expression of PABPN1 in non-muscle invasive/muscle invasive BC samples from GSE13507. (G) Relative expression of PABPN1 in samples from TCGA BLCA without/with lymph node metastases. Data are presented as the mean ± SD of three independent experiments. * P < 0.05.

**Additional file 2: Figure S2**. Upregulated PABPN1 leads to more transcripts with longer 3’UTRs in BC cells. (A) The long transcript-to-total transcript ratios of indicated genes upon PABPN1 overexpression or knockdown measured by qRT-PCR. (B) 3’UTR profiles of PTCD1, AACS, ATP6AP2, PIGG, CDCA2, TMEM97, ZNF777, REPIN1, and CCND3. (C) Diagram showing vectors containing wild-type or mutant 3’UTRs of PABPN1 target genes. (D) Binding capacities of PABPN1 to wild-type or mutant 3’UTRs evaluated by RIP assays with anti-PABPN1 antibody. IgG was used as a negative control. (E) Pull‑down assay with RNA probes targeting mRNA of AACS, PIGG, and CDCA2 verifying the interaction between PABPN1 and its targets. Oligo probe was used as a negative control. Data are presented as the mean ± SD of three independent experiments. * P < 0.05.

**Additional file 3: Figure S3**. PABPN1 regulates the 3’UTR lengths of ATP6AP2, TMEM97, CDCA2, CCND3, and AACS. (A) Flow cytometric analysis showing the cell cycle distribution of cells stably transfected with CDCA2 or CCND3 overexpressing vectors. (B) The neutral lipids content in cells stably transfected with AACS overexpressing vector. Neutral lipids were stained with BODIPY 493/503 dye (green). Nuclei were stained with Hoechst (blue). 200×. (C) Proliferation of BC cells shown by colony formation assay. (D) Representative pictures of migrating and invading cells analyzed by Transwell assay. 200×. Data are presented as the mean ± SD of three independent experiments. * P < 0.05.

**Additional file 4: Table S1**. 94 potential APA regulators.

**Additional file 5: Table S2**. The sequences of primers used in this study.

## Data Availability

The datasets used and/or analysed during the current study are available from the corresponding author on reasonable request.

## Abbreviations

BC: Bladder cancer
APA: Alternative polyadenylation
PASs: Polyadenylation signals
CPSF: Polyadenylation specificity factors
CSTFs: Cleavage stimulation factors
PABPN1: Poly(A) binding protein nuclear 1
3’UTR: 3’ Untranslated region
OPMD: Oculopharyngeal muscular dystrophy
DSB: Double-strand breaks
CCK-8: Cell counting kit-8
RIP: RNA binding protein immunoprecipitation
qRT-PCR: Quantitative real-time PCR
3’RACE: Rapid amplification of cDNA 3’ ends
DaPars: Dynamic analysis of alternative polyadenylation from RNA-Seq
RNA-Seq: RNA sequencing
TCGA: The Cancer Genome Atlas
GEO: Gene Expression Omnibus
DEGs: Differentially expressed genes
