# Supplementary material for: PABPN1 regulates mRNA alternative polyadenylation to inhibit bladder cancer progression
Source: Cell Biosci. 2023 Mar 6;13:45. doi: 10.1186/s13578-023-00997-6 (PMC9987104; doi:10.1186/s13578-023-00997-6)
Supplement: Supplementary file 1 — Additional file 1: Figure S1. Potential APA regulator PABPN1 is downregulated in BC cells. (A) Venn diagrams showing 41 potential 3’UTR-lengthen regulators in BC. (B) Venn diagrams showing 53 potential 3’UTR-shorten regulators in BC. (C) PABPN1 mRNA levels in human normal urothelial epithelial cell line and BC cell lines. (D) PABPN1 mRNA levels in BC tissues (T) and adjacent non-tumorous tissues (N). (E) Relative expression of PABPN1 in low/high grade BC samples from GSE13507. (F) Relative expression of PABPN1 in non-muscle invasive/muscle invasive BC samples from GSE13507. (G) Relative expression of PABPN1 in samples from TCGA BLCA without/with lymph node metastases. Data are presented as the mean ± SD of three independent experiments. * P < 0.05. [file 13578_2023_997_MOESM1_ESM.docx]

**
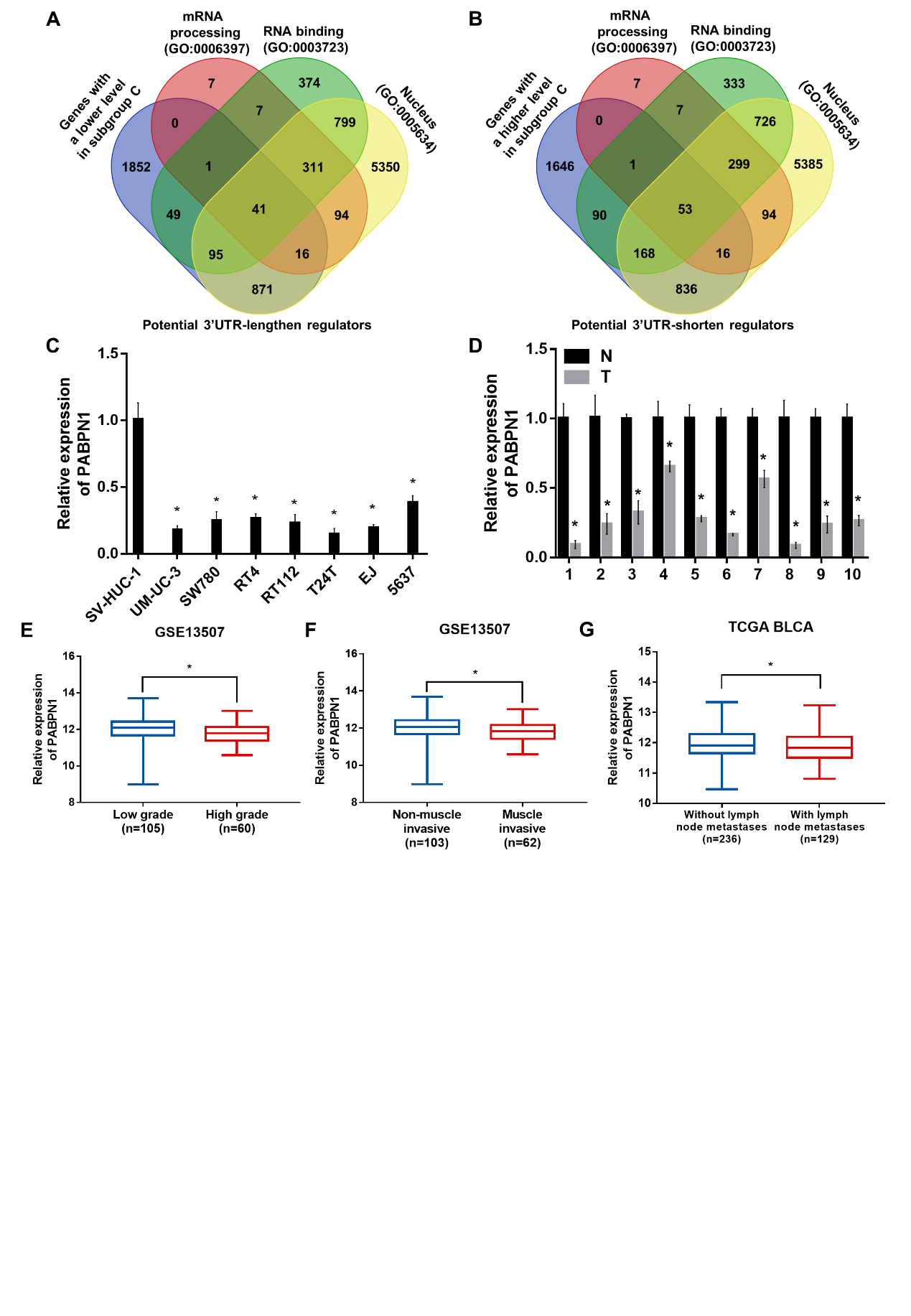
Figure S1. Potential APA regulator PABPN1 is downregulated in BC cells. (A)** Venn diagrams showing 41 potential 3’UTR-lengthen regulators in BC. **(B)** Venn diagrams showing 53 potential 3’UTR-shorten regulators in BC. **(C)** PABPN1 mRNA levels in human normal urothelial epithelial cell line and BC cell lines. **(D)** PABPN1 mRNA levels in BC tissues (T) and adjacent non-tumorous tissues (N). **(E)** Relative expression of PABPN1 in low/high grade BC samples from GSE13507. **(F)** Relative expression of PABPN1 in non-muscle invasive/muscle invasive BC samples from GSE13507. **(G)** Relative expression of PABPN1 in samples from TCGA BLCA without/with lymph node metastases. Data are presented as the mean ± SD of three independent experiments. * P < 0.05.
