# Supplementary material for: PABPN1 regulates mRNA alternative polyadenylation to inhibit bladder cancer progression
Source: Cell Biosci. 2023 Mar 6;13:45. doi: 10.1186/s13578-023-00997-6 (PMC9987104; doi:10.1186/s13578-023-00997-6)
Supplement: Supplementary file 2 — Additional file 2: Figure S2. Upregulated PABPN1 leads to more transcripts with longer 3’UTRs in BC cells. (A) The long transcript-to-total transcript ratios of indicated genes upon PABPN1 overexpression or knockdown measured by qRT-PCR. (B) 3’UTR profiles of PTCD1, AACS, ATP6AP2, PIGG, CDCA2, TMEM97, ZNF777, REPIN1, and CCND3. (C) Diagram showing vectors containing wild-type or mutant 3’UTRs of PABPN1 target genes. (D) Binding capacities of PABPN1 to wild-type or mutant 3’UTRs evaluated by RIP assays with anti-PABPN1 antibody. IgG was used as a negative control. (E) Pull‑down assay with RNA probes targeting mRNA of AACS, PIGG, and CDCA2 verifying the interaction between PABPN1 and its targets. Oligo probe was used as a negative control. Data are presented as the mean ± SD of three independent experiments. * P < 0.05. [file 13578_2023_997_MOESM2_ESM.docx]

**
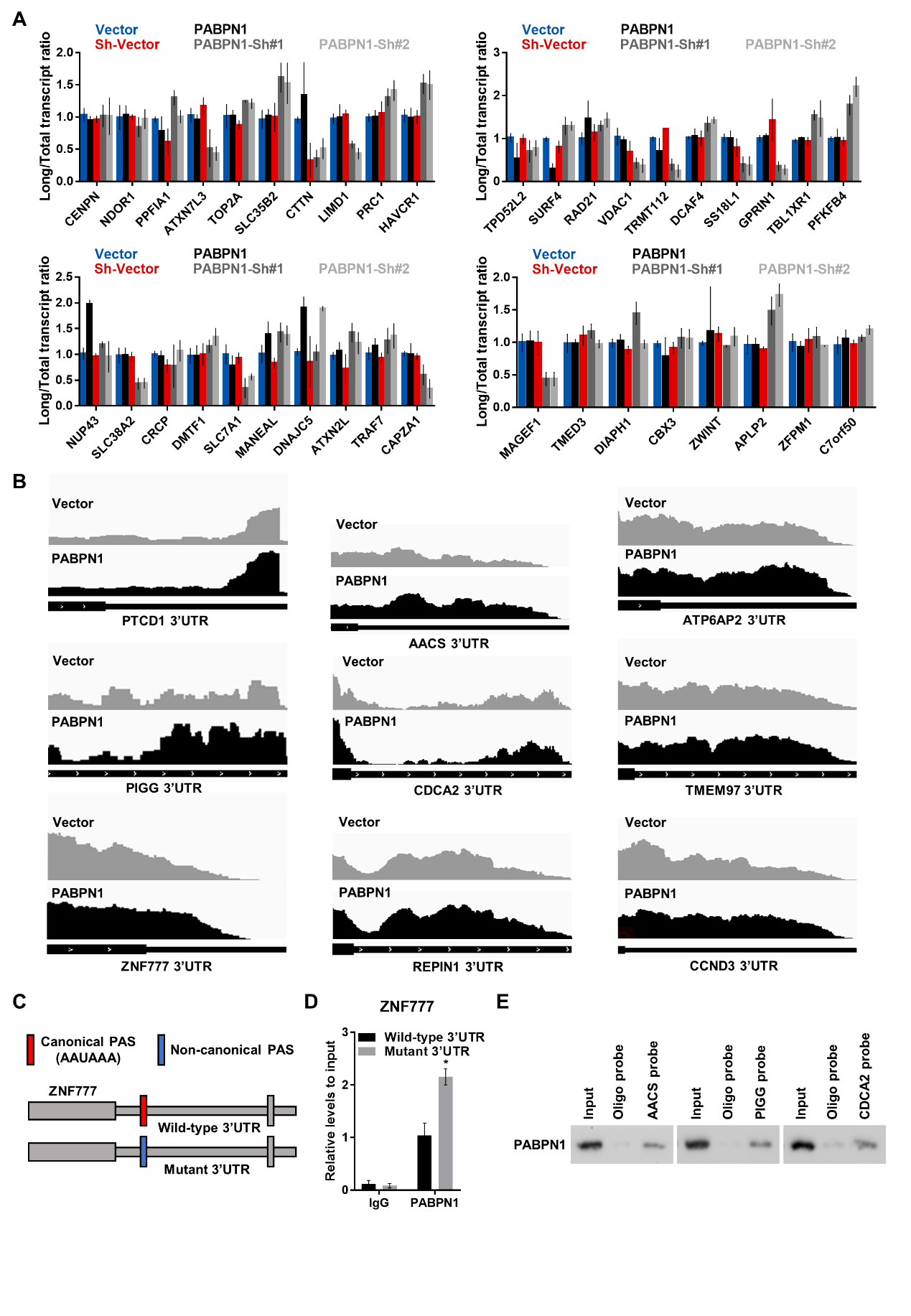
Figure S2. Upregulated PABPN1 leads to more transcripts with longer 3’UTRs in BC cells. (A)** The long transcript-to-total transcript ratios of indicated genes upon PABPN1 overexpression or knockdown measured by qRT-PCR. **(B)** 3’UTR profiles of PTCD1, AACS, ATP6AP2, PIGG, CDCA2, TMEM97, ZNF777, REPIN1, and CCND3. **(C)** Diagram showing vectors containing wild-type or mutant 3’UTRs of PABPN1 target genes. **(D)** Binding capacities of PABPN1 to wild-type or mutant 3’UTRs evaluated by RIP assays with anti-PABPN1 antibody. IgG was used as a negative control. **(E)** Pull‑down assay with RNA probes targeting mRNA of AACS, PIGG, and CDCA2 verifying the interaction between PABPN1 and its targets. Oligo probe was used as a negative control. Data are presented as the mean ± SD of three independent experiments. * P < 0.05.
