# Supplementary material for: PABPN1 regulates mRNA alternative polyadenylation to inhibit bladder cancer progression
Source: Cell Biosci. 2023 Mar 6;13:45. doi: 10.1186/s13578-023-00997-6 (PMC9987104; doi:10.1186/s13578-023-00997-6)
Supplement: Supplementary file 3 — Additional file 3: Figure S3. PABPN1 regulates the 3’UTR lengths of ATP6AP2, TMEM97, CDCA2, CCND3, and AACS. (A) Flow cytometric analysis showing the cell cycle distribution of cells stably transfected with CDCA2 or CCND3 overexpressing vectors. (B) The neutral lipids content in cells stably transfected with AACS overexpressing vector. Neutral lipids were stained with BODIPY 493/503 dye (green). Nuclei were stained with Hoechst (blue). 200×. (C) Proliferation of BC cells shown by colony formation assay. (D) Representative pictures of migrating and invading cells analyzed by Transwell assay. 200×. Data are presented as the mean ± SD of three independent experiments. * P < 0.05. [file 13578_2023_997_MOESM3_ESM.docx]

**
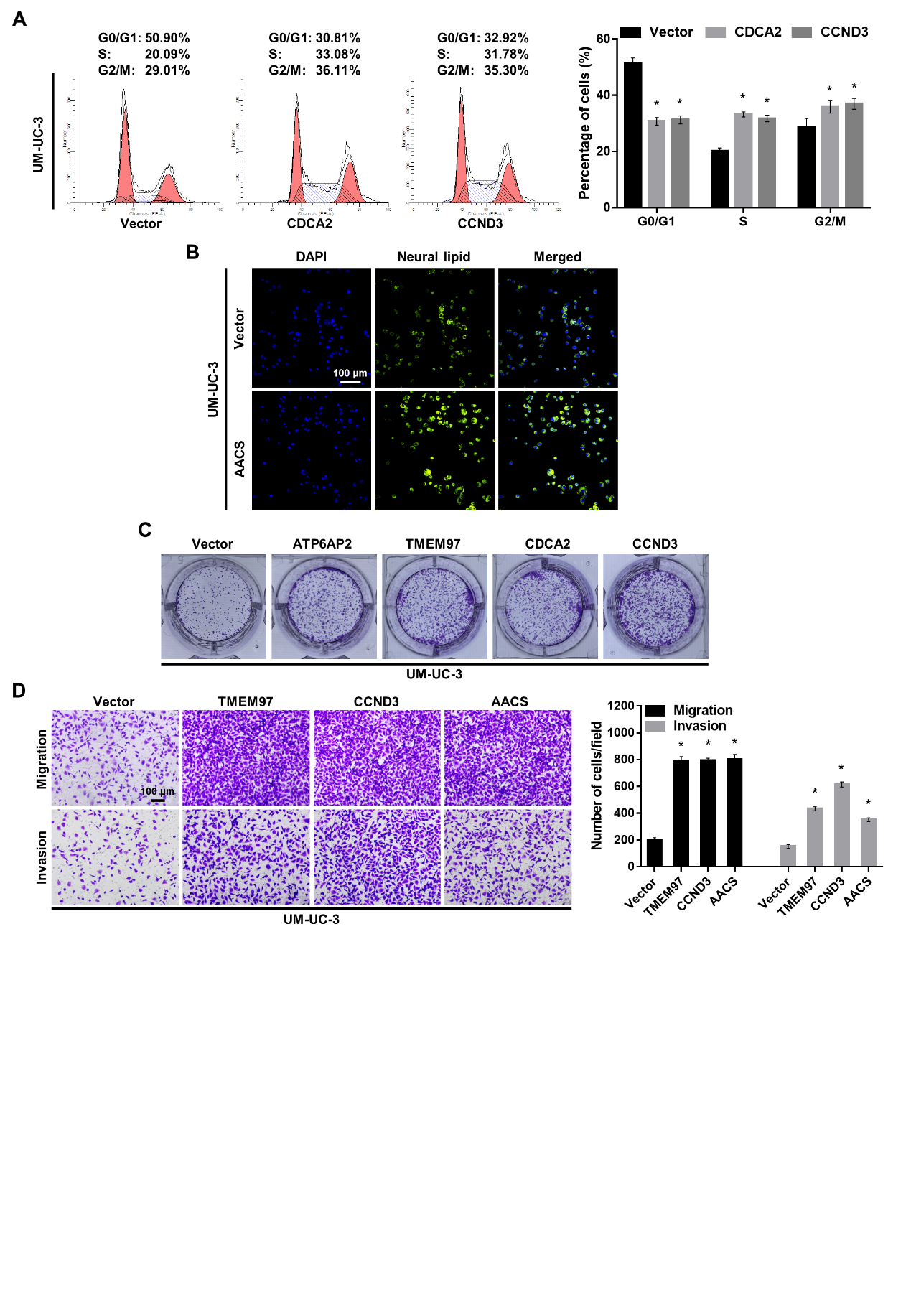
Figure S3. PABPN1 regulates the 3’UTR lengths of ATP6AP2, TMEM97, CDCA2, CCND3, and AACS. (A)** Flow cytometric analysis showing the cell cycle distribution of cells stably transfected with CDCA2 or CCND3 overexpressing vectors. **(B)** The neutral lipids content in cells stably transfected with AACS overexpressing vector. Neutral lipids were stained with BODIPY 493/503 dye (green). Nuclei were stained with Hoechst (blue). 200×. **(C)** Proliferation of BC cells shown by colony formation assay. **(D)** Representative pictures of migrating and invading cells analyzed by Transwell assay. 200×. Data are presented as the mean ± SD of three independent experiments. * P < 0.05.
